# Surgical management of post-corrosive gastric outlet obstruction: Outcome assessment in adults and pediatric patients in resource-limited settings

**DOI:** 10.5339/qmj.2025.74

**Published:** 2025-08-12

**Authors:** Anas Mohammed Shuwail, Ali Lotf Al-Amry, Yasser Abdurabo Obadiel, Mohammed Mohammed Alsurmi, Afaf Mohammed Al-Dhubaibi, Jalal Mohammed Al-Hubaishi, Haitham Mohammed Jowah

**Affiliations:** 1 Department of Surgery, Al-Thawra Modern General Hospital, Sana’a City, Yemen; 2 Department of Surgery, Amran University, Amran, Yemen; 3 Department of Surgery, Faculty of Medicine and Health Science, Sana’a University, Sana’a City, Yemen *Email: h.jowah@su.edu.ye

**Keywords:** Corrosive injuries, gastric outlet obstruction, surgical management, outcomes, Yemen

## Abstract

**Objective:**

This study aims to evaluate the outcomes of surgical treatment in adult and pediatric patients with post-corrosive gastric outlet obstruction (GOO) in Yemen.

**Patients and methods:**

A prospective observational study was conducted at Al-Thawra Modern General Hospital (TMGH), from January 1, 2019 to January 31, 2023. The study involved 77 patients, comprising both adults and children, who were admitted for surgical treatment of post-corrosive GOO. Data were collected from medical records, patient interviews, and follow-up visits.

**Results:**

Among the 77 patients, 77% (*n* = 59) were pediatric patients and 23% (*n* = 18) were adults. The mean age of the cohort was 10.6 years, with an average age of 4.12 years for pediatric patients and 30.8 years for adults. The primary cause of post-corrosive GOO was the accidental ingestion of acidic substances, accounting for 97% of cases. Common symptoms at presentation included vomiting, early satiety, and weight loss. Definitive surgery was performed in one stage in 95% of patients, with Heineke–Mikulicz pyloroplasty being the most common procedure (82%) in pediatric cases, whereas gastrojejunostomy was mainly used in adults (67%). Postoperative complications occurred in 22% of patients, with vomiting, wound infection, and aspiration pneumonia being the most common. Anastomotic restriction was observed in two pediatric patients who required reoperation. The overall mortality rate was 1.3%, with one pediatric patient having succumbed. Notably, improvements in GOO symptoms were observed in 96% of patients. Pediatric patients had a longer median hospital stay (6.5 days) than adult patients (6 days).

**Conclusion:**

Corrosive injuries, particularly in the pediatric population, pose a significant issue in Yemen due to the unsafe storage of sulfuric acid. It is recommended to conduct early surgical interventions within 4 weeks after ingestion to prevent weight loss and to reduce prolonged hospitalization. Further research and interventions are needed to prevent such injuries, improve public awareness, and regulate the sale and storage of corrosive substances.

## 1. INTRODUCTION

Corrosive injuries to the upper gastrointestinal (GI) tract represent a major public health challenge, particularly in low- and middle-income countries where unsafe storage practices and limited healthcare resources exacerbate the problem.^1^ These injuries often lead to severe complications, including gastric outlet obstruction (GOO), which disproportionately affects vulnerable populations, such as children. In Yemen, the ongoing military conflict has led to widespread electricity shortages, forcing communities to rely heavily on solar energy sources. Sulfuric acid from these batteries is often stored in unsafe containers, posing a significant risk of accidental ingestion, particularly among children.^2^ Such hazardous practices highlight the urgent need for preventive measures and improved public awareness campaigns.

The extent and severity of corrosive injuries depend on several factors, including the type of agent, the amount ingested, and the duration of contact. Alkalis cause liquefaction necrosis, leading to deep tissue penetration and esophageal damage, whereas acids result in coagulative necrosis, primarily affecting the stomach.^3^ A common physiological response to corrosive ingestion is pylorospasm, which prolongs the contact time between acids and the gastric mucosa, resulting in severe fibrosis and eventual GOO.^4,5^

Gastric damage often affects the antrum or distal stomach, primarily due to the accumulation of corrosive agents in this region. This pooling is often the result of pylorospasm, which prolongs the contact time with the corrosive substance, leading to significant harm.^6^ Such damage produces severe fibrosis, ultimately resulting in strictures and GOO. It is crucial to address this issue to prevent further complications and ensure effective treatment.

Despite advances in surgical techniques, the optimal timing and type of intervention for post-corrosive GOO remain controversial.^7^ Some authors advocate delayed repair, arguing that maximum fibrosis develops over time, while others recommend early surgical intervention to prevent complications such as weight loss and prolonged hospitalization. This debate underscores the need for evidence-based guidelines tailored to resource-limited settings.

Although previous studies have explored surgical outcomes for post-corrosive GOO, only a few have addressed the unique challenges encountered in resource-limited settings where access to advanced diagnostic tools and multidisciplinary care is limited. Moreover, there is a paucity of data comparing outcomes between pediatric and adult patients. The objective of our study was to fill these gaps by evaluating the outcomes of surgical management for post-corrosive GOO in both populations. By assessing factors such as age distribution, types of corrosive agents ingested, time of presentation, surgical approaches used, and associated complications, we aim to shed light on the unique aspects of this condition in our setting.

## 2. PATIENTS AND METHODS

### 2.1. Study design

This prospective observational study evaluated the outcomes of surgical management for post-corrosive GOO in pediatric and adult patients. The primary objective of this study was to assess the effectiveness of early surgical intervention and to identify the factors that influence patient outcomes.

### 2.2. Study setting and population

The study was conducted at Al-Thawra Modern General Hospital (TMGH) between January 1, 2019 and January 31, 2023. A total of 77 patients, comprising adults and children, who were admitted for surgical treatment of post-corrosive GOO were included in the study. Inclusion criteria were a confirmed diagnosis of post-corrosive GOO and patients who underwent surgical intervention. Exclusion criteria included patients with severe comorbid conditions (e.g., advanced cardiovascular disease, renal failure), those managed conservatively or initially treated outside our hospital, and those undergoing surgery for esophageal stricture without evidence of GOO.

The sample size of 77 patients was determined based on the number of eligible patients admitted during the study period. Although formal power calculations were not performed, the sample size was deemed adequate based on similar studies conducted in resource-limited settings that used comparable sample sizes to evaluate surgical outcomes for post-corrosive injuries.

### 2.3. Preoperative preparation

All patients underwent thorough preoperative preparation, including the correction of intravenous fluids, electrolytes, and albumin levels. Nutritional support was provided as needed, with total parenteral nutrition or feeding jejunostomy administered in cases of severe malnutrition. Upper GI endoscopy was performed by experienced surgeons under general anesthesia to assess the extent of injury and to guide surgical planning. ENT (Ear, Nose, and Throat) evaluations were not routinely conducted due to delayed presentations and limited documentation from primary healthcare centers. Precautions were taken during the endoscopy to minimize the risk of iatrogenic perforation.

### 2.4. Surgical interventions

Surgical procedures were tailored to the extent of gastric involvement and the surgeon’s expertise. The following interventions were performed:

1. **Heineke–Mikulicz pyloroplasty:** This procedure, involving a longitudinal incision across the pylorus that is closed transversely, is predominantly used in pediatric patients and occasionally in adults. A longitudinal incision measuring 4 cm, centered on the pylorus, was closed transversely using interrupted 3.0 sutures with full-thickness bites.
2. **Gastrojejunostomy:** The anastomosis between the stomach and the jejunum was primarily performed in adult patients with gastric shrinkage or complete pyloric obstruction.
3. **Total gastrectomy with Roux-en-Y reconstruction:** This procedure, which involves the complete removal of the stomach followed by Roux-en-Y reconstruction, was performed in adult patients with severe gastric shrinkage and cachexia due to late presentation.
4. **Feeding jejunostomy:** This supportive procedure involves the placement of a feeding tube into the jejunum to provide enteral nutrition for patients who require preoperative or postoperative nutritional support due to severe malnutrition or delayed oral feeding.

### 2.5. Data collection

Data were collected through the review of medical records, interviews with patients, and subsequent follow-up visits. The variables assessed included:

- **Demographic characteristics:** Age, sex, and comorbidities (e.g., hypertension, diabetes).
- **Details of corrosive ingestion:** The type of corrosive agents (acidic, alkaline, or other) and the intent behind ingestion (accidental or suicidal).
- **Diagnostic findings:** Results from endoscopic examinations and barium meal studies (e.g., pyloric obstruction, gastric dilation, or shrinkage).
- **Surgical procedures:** Various types of interventions that were performed.
- **Postoperative outcomes:** Complications (e.g., wound infection, anastomotic leakage), length of hospital stay (LOS), mortality, and improvement in GOO symptoms (as assessed by the absence of vomiting and weight gain).

### 2.6. Outcome measures

The primary outcome assessed was the success of surgical management, which was evaluated based on the analysis of postoperative complications, mortality rates, LOS, and symptom improvement. The secondary objectives included the prevalence of GOO, the types of corrosive agents ingested, and time to presentation.

### 2.7. Statistical analysis

Descriptive statistics were used to summarize the data. Continuous variables are expressed as means±standard deviation (SD) or medians with interquartile ranges (IQR), depending on their distribution. Categorical variables are presented as frequencies and percentages. Statistical analyses were performed using IBM SPSS Statistics (v.26). For inferential analysis, appropriate statistical tests (e.g., chi-square test for categorical variables and t-test or Mann–Whitney U test for continuous variables) were used to compare outcomes between groups. A *p* value of < 0.05 was considered statistically significant.

## 3. RESULTS

A total of 77 patients were included in the study, with pediatric patients (*n* = 59) constituting 77% of the cohort and adults (*n* = 18) accounting for the remaining 23%. The overall mean age was 10.6 years, with an average age of 4.12 years for pediatric patients and 30.8 years for adults. Males predominated in the pediatric group (70%), whereas females were predominant in the adult group (78%). Comorbidities were present in 5% of patients, with a slightly higher rate in adults (11%) (Table 1).

The majority of patients (97%) ingested acidic substances, primarily sulfuric acid from solar batteries, as a result of unsafe storage practices. In contrast, alkaline substances, such as flash cleaner, were involved in only 3% of cases, resulting in esophageal strictures in two pediatric patients who required endoscopic dilation. Ingestion was accidental in 97% of cases, with intentional ingestion observed in only two adult patients. For adults, accidental ingestion often occurs because of unsafe storage practices, particularly sulfuric acid from solar batteries (Table 2).

Common symptoms at presentation included vomiting, early satiety, and weight loss. Abdominal pain and fever were rare (<6%). Laboratory investigations revealed anemia in 54.6% of patients, hypokalemia in 70.1%, and hypoalbuminemia in 50.7%. Endoscopic findings revealed complete pyloric obstruction in 77% of patients and partial obstruction in 23% of patients. Barium meal studies indicated a dilated stomach in 87% of patients, partial shrinkage in 10.4%, and complete shrinkage in 2.6%. However, detailed endoscopic or radiological classifications using standardized grading scales were not feasible due to limited documentation in the medical records.

Definitive repair was performed in one stage in 95% of patients, with the majority (46.7%) undergoing surgery at 4 weeks post-ingestion. The decision for early surgical intervention within this timeframe was made to prevent further weight loss and reduce hospital stay by enabling earlier oral feeding. All pediatric patients (*n* = 59) underwent Heineke–Mikulicz pyloroplasty, a procedure involving a longitudinal incision across the pylorus that is closed transversely. This approach was associated with satisfactory outcomes in all cases, defined by the resolution of GOO symptoms (absence of vomiting), weight gain, and no need for reoperation. Several procedures were performed in adult patients, including gastrojejunostomy in 12 patients (67%) and total gastrectomy with Roux-en-Y reconstruction in two patients (11%) (Table 3).

Postoperative complications occurred in 22% of patients, with the most prevalent being vomiting (10.4%), wound infection (6.5%), and aspiration pneumonia (6.5%). Anastomotic restriction occurred in two pediatric patients who required reoperation. Mortality was recorded in one pediatric patient (1.3%). An improvement in GOO symptoms, assessed clinically by the absence of vomiting and weight gain, was observed in 96% of patients. The median LOS was 6.5 days for pediatric patients and 6 days for adults (Table 4).

## 4. DISCUSSION

This prospective observational study was conducted at Al-Thawra Modern General Hospital (TMGH) from January 1, 2019 to January 31, 2023. A total of 77 patients were included in the study, comprising 45 (58.4%) males and 32 (41.6%) females. The majority (77%) of patients were pediatric, with 59 cases involving children under the age of 5, consistent with the findings reported by Nasr et al.^11^ The remaining 18 (23%) cases were adults, with a mean age of 30.8 ± 9.147 years, consistent with the data reported by Gupta et al.^12^ The relatively small number of adult cases may be attributed to the rarity of such injuries in this population.^13^

In our study, 75 patients presented with a history of accidental corrosive ingestion, primarily involving sulfuric acid from batteries due to unsafe storage practices, consistent with the findings of Elhalaby et al.^8^ Accidental ingestion is the primary cause of corrosive injuries in both pediatric and adult populations. For adults, the accidental ingestion of sulfuric acid from solar batteries often occurs because of unsafe storage practices. This finding highlights the importance of public awareness campaigns and stricter regulations for the storage of corrosive substances.

A total of 40 (51.9%) patients had an initial latent period of 1–2 weeks after ingestion, during which they sought medical advice at rural hospitals or centers, but did not fully recover until 3–4 weeks later when they presented to our hospital. The remaining 37 (48.1%) patients did not initially seek medical attention due to factors such as poverty, illiteracy, or the distance to healthcare centers, presenting only after they had developed complete pyloric obstruction. GOO can develop as early as 7 days or as late as 6 years after ingestion, depending on the extent of the initial injury.^1^ In rural hospitals, the initial conservative management of corrosive ingestion typically involves supportive measures such as intravenous fluids and proton pump inhibitors to mitigate acute symptoms. However, the lack of standardized national protocols for managing corrosive injuries in Yemen, particularly in resource-constrained rural areas, often leads to delayed diagnosis and referral. This gap highlights the need for training healthcare providers and establishing clear guidelines to ensure the timely recognition and referral of patients with potential GOO.

Upon presentation to the hospital, all patients reported symptoms such as vomiting, early satiety, and weight loss, with a scaphoid abdomen and a dilated stomach, except for 10 adult patients who had a shrunken stomach on percussion. Laboratory investigations revealed anemia in 54.6% of patients, hypokalemia in 70.1%, and hypoalbuminemia in 50.7%, similar to the findings reported by Lebeau et al.^14^ All patients underwent barium studies and upper GI endoscopy, which revealed a dilated stomach in 87% of cases, partial shrinkage in 10.4%, and complete shrinkage in 2.6%. Complete pyloric obstruction was observed in 76.6% of patients, whereas partial obstruction was noted in 23.4%. Two pediatric patients had esophageal strictures due to alkaline ingestion and required feeding jejunostomy and serial esophageal dilatation, followed by Heineke–Mikulicz pyloroplasty.

Gastric damage often affects the antrum or distal stomach, primarily due to the accumulation of corrosive agents in this region. This pooling is often the result of pylorospasm, which prolongs the contact time of the corrosive substance, leading to significant harm. Such damage initiates a cascade of pathological changes, including severe fibrosis, ultimately resulting in strictures and GOO. Early surgical intervention during the proliferation phase of scarring (3–21 days) is crucial to prevent irreversible fibrotic changes and to ensure optimal outcomes. Understanding these mechanisms underscores the importance of timely surgical intervention to mitigate the progression of fibrosis and improve patient outcomes.^15,16^

The surgical approach depends on the site and extent of gastric involvement, and the optimal timing and type of surgery are controversial. Chaudhary et al. advocated a delayed repair, arguing that the fibrotic process occurs over time, with maximum fibrosis developing before surgery.^9^ However, we preferred early repair for the majority of our patients (64.9%) within the first 3–4 weeks after ingestion, following the correction of fluid and electrolyte disturbances. Early surgical intervention prevents further weight loss and prolonged hospital stay by enabling earlier oral feeding, which is consistent with the findings reported by Ray et al. and Tekant et al.^6,10^ A study conducted by Meena et al. highlighted that the management of corrosive injuries of the upper GI tract remains a major surgical dilemma.^17^

All pediatric patients (*n* = 59) underwent Heineke–Mikulicz pyloroplasty, yielding satisfactory results, characterized by the resolution of vomiting, weight gain, and minimal major complications, which is consistent with the findings of Elhalaby et al.^8^ Heineke–Mikulicz pyloroplasty involves a longitudinal incision across the pylorus that is closed transversely, making it particularly effective in pediatric patients with post-corrosive GOO. Several procedures were performed in adult patients. Two (2.6%) patients with complete gastric shrinkage underwent total gastrectomy with Roux-en-Y esophagojejunostomy after 4 months of jejunostomy feeding because of late presentation and severe cachexia. Thirteen (16.9%) patients underwent gastrojejunostomy – eight due to gastric shrinkage and five due to complete pyloric obstruction and the surgeon’s preference – which is consistent with the findings of Nasr et al., Ceylan et al., and Ayyaz et al.^11,18,19^ Three adult patients underwent Heineke–Mikulicz pyloroplasty, yielding good outcomes. The choice of procedure depended on the gastric segments as well as the surgeon’s experience and preferences. Similarly, El-Asmar and Allam performed two total gastrectomies, two gastrojejunostomies, and nine Heineke–Mikulicz pyloroplasties, among other procedures.^20^

Postoperative feeding was initiated 4–5 days postoperatively with liquids, gradually advancing to soft, semisolid, and solid diets, as described by Ali et al.^21^ Postoperative complications included superficial surgical site infections in five (6.5%) patients treated with dressing changes alone, consistent with the findings of Gupta et al. and Prasad and Patel.^12,22^ Aspiration pneumonia occurred in six (7.8%) patients, comprising four pediatric and two adult cases, primarily due to aspiration during intubation, with three patients requiring ICU admission for 1–2 days, while the remaining three patients were managed in the ward, similar to the findings of Gupta et al.^12^ Vomiting was observed in eight (10.4%) patients, with three experiencing postoperative nausea and vomiting that improved with the administration of antiemetics within a week. One pediatric male and one adult female patient who underwent Heineke–Mikulicz pyloroplasty, along with one adult female who underwent gastrojejunostomy, developed vomiting 2–5 weeks postoperatively, likely due to symptomatic bile reflux gastritis or prolonged gastric dilatation and gastritis. These cases showed improvement with the use of domperidone, proton pump inhibitors, or H2 blockers, consistent with the findings of Shetty et al.^23^ Additionally, two (2.6%) pediatric patients who underwent Heineke–Mikulicz pyloroplasty presented with vomiting due to anastomotic site restriction – one at 3 weeks postoperatively and the other at 6 months postoperatively – both of whom underwent reoperation with Heineke–Mikulicz pyloroplasty, similar to the findings reported by Ceylan et al. and El-Asmar et al.^18,20^

One (1.3%) patient died of aspiration after surgery. The mean length of the postoperative hospital stay was 6.57 ± 0.979 days, ranging from 5 to 9 days, which is consistent with the findings of Prasad and Patel.^22^ Both pediatric and adult patients exhibited an acceptable increase in postoperative weight.

Several limitations of our study must be acknowledged. First, the lack of detailed endoscopic or radiological classifications using standardized grading scales (e.g., Zargar’s classification) limited our ability to comprehensively assess the severity of injuries. Future studies should incorporate such tools to guide tailored interventions. Second, the single-center nature of the study may limit the generalizability of the findings, particularly to other settings with limited resources. Multicenter studies involving diverse populations are needed to validate our results. Finally, the relatively short follow-up period (median duration: 6 months) restricted our ability to evaluate long-term outcomes, such as the recurrence of strictures or nutritional deficiencies. Longer follow-up durations are essential to fully understand the impact of surgical interventions.

The high incidence of accidental ingestion observed in our study underscores the urgent need for preventive measures. Implementing stricter regulations on the sale and storage of corrosive substances, particularly sulfuric acid from solar batteries, is essential to reduce the incidence of such injuries. Community awareness campaigns targeting safe storage practices could play a pivotal role in preventing accidental ingestion among vulnerable pediatric populations.

## 5. CONCLUSION

Corrosive injuries to the upper GI tract pose a significant health challenge in Yemen, particularly among children. Effective management, including early surgical intervention within 3–4 weeks after ingestion, is essential for improving patient outcomes. Additionally, preventive measures, such as public awareness campaigns and stricter regulations on the storage of corrosive substances, are crucial to reduce the incidence of such injuries. Further research is needed to develop effective prevention and management strategies for resource-poor settings.

## LIST OF ABBREVIATIONS

**Table:** 

GI	Gastrointestinal
GOO	Gastric Outlet Obstruction
ICU	Intensive Care Unit
IQR	Interquartile Range
LOS	Length of Hospital Stay
TMGH	Al-Thawra Modern General Hospital

## ETHICS APPROVAL AND INFORMED CONSENT

Ethical approval for this study was obtained from the Ethical Committee of Sana’a University, Yemen (IRB Approval Number: SU-2019/77). Written informed consent was obtained from all participants, ensuring their participation was entirely voluntary. Participants were informed that their data would be used solely for research purposes and that strict confidentiality would be maintained. This study adhered to the ethical principles outlined in the Declaration of Helsinki.

## COMPETING INTERESTS

The authors have no conflicts of interest to declare.

## AUTHORS’ CONTRIBUTIONS

AMS: Contributed to the study concept, data collection, and the initial drafting of the manuscript. ALA: Participated in the study design, surgical interventions, and the critical revision of the manuscript. YAO: Conducted data analysis and interpretation, and contributed to the drafting of the discussion section. MMA: Supervised the study methodology and assisted in patient recruitment and follow-up. AMA: Managed the literature review and editing assistance for the final version of the manuscript. JMH: Coordinated with the institutional ethics committee and facilitated access to medical records. HMJ: Led the overall coordination of the study, statistical review, and correspondence with the journal. All authors read and approved the final manuscript.

## Figures and Tables

**Table 1.** Demographic characteristics of the study groups.

Demographic characteristics	Pediatric (*n* = 59)	Adult (*n* = 18)	Total (*n* = 77)
Age (years), mean ± SD	4.12 ± 2.15	30.8 ± 9.14	10.6 ± 12.37
Gender			
Male	41 (70%)	4 (22%)	45 (58%)
Female	18 (3.5%)	14 (78%)	32 (42%)
Comorbidity	3 (5%)	2 (11%)	5 (6.5%)

**Table 2.** Preoperative characteristics of the study groups.

Variables	Pediatric (*n* = 59)	Adult (*n* = 18)	Total (*n* = 77)
Intention for corrosive ingestion			
Accidently	59 (100%)	16 (89%)	75 (97%)
Suicidal	0	2 (11%)	2 (3%)
Type of ingested substance			
Acidic	57 (97%)	18 (100%)	75 (97%)
Alkali	2 (3%)	0	2 (3%)
Presenting symptoms			
Vomiting	59 (100%)	18 (100%)	77 (100%)
Early satiety	59 (100%)	18 (100%)	77 (100%)
Weight loss	59 (100%)	18 (100%)	77 (100%)
Abdominal pain	1 (2%)	1 (6%)	2 (3%)
Fever	2 (3%)	1 (6%)	3 (4%)
Anemia	29 (49%)	13 (72%)	42 (54.5%)
Hypokalemia	40 (68%)	14 (78%)	54 (70%)
Hypoalbuminemia	27 (46%)	12 (67%)	39 (51%)
Time from ingestion to presentation			
≤1 month	39 (66%)	11 (61%)	50 (65%)
>1 month	20 (34%)	7 (39%)	27 (35%)
Associated esophageal stricture	2 (3%)	0	2 (3%)
Endoscopy findings			
Complete obstruction	44 (75%)	15 (83%)	59 (77%)
Partial obstruction	15 (25%)	3 (17%)	18 (23%)
Barium study findings			
Dilated stomach	55 (93%)	12 (67%)	67 (87%)
Shrinkage	4 (7%)	6 (33%)	10 (13%)

**Table 3.** Surgical management of the study groups.

Variables	Pediatric (*n* = 59)	Adult (*n* = 18)	Total (*n* = 77)
Operation stages			
One stage	57 (95%)	16 (94%)	73 (95%)
Two stages	3 (5%)	1 (6%)	4 (5%)
Definitive surgical procedure			
Heineke–Mikulicz pyloroplasty	59 (100%)	4 (22%)	63 (82%)
Gastrojejunostomy	0	12 (67%)	12 (16%)
Total gastrectomy with Roux-en-Y	0	2 (11%)	2 (3%)
Time to definitive repair			
3 weeks	14 (24%)	0	14 (18.2%)
4 weeks	27 (46%)	9 (50%)	36 (46.7%)
5 weeks	9 (15%)	5 (28%)	14 (18.2%)
6 weeks	5 (8.5%)	2 (11%)	7 (9.1%)
More than 6 weeks	4 (7%)	2 (11%)	6 (7.8%)

**Table 4.** Postoperative complications and outcomes among the study groups.

Variables	Pediatric (*n* = 59)	Adult (*n* = 18)	Total (*n* = 77)
Postoperative complications	11 (17%)	6 (33%)	17 (22%)
Vomiting	4 (36.4%)	4 (67%)	8 (47%)
Wound infection	3 (27%)	2 (33%)	5 (29%)
Aspiration pneumonia	4 (36%)	2 (33%)	6 (35%)
Anastomotic site restriction	2 (18%)	0	2 (11%)
Mortality	1 (9%)	0	1 (6%)
Postoperative outcomes			
Improved	56 (95%)	18 (100%)	74 (96%)
Not improved	3 (5%)	0	3 (4%)
LOS (median, IQR)	6 (5–9)	30 (19–48)	

LOS: Length of hospital stay, IQR: Interquartile range.
